# The Division of Fees

**Published:** 1910-05

**Authors:** John H. Pryor, Grover W. Wende, Bernard Bartow, Edwin A. Bowerman, Irying P. Lyon, Matthew D. Mann, F. Park Lewis, Thomas F. Welsh, William Gaertner

**Affiliations:** Chairman; Ex-officio; Secretary


### CORRESPONDENCE.

---

### The Division of Fees[1]

*Editor Buffalo Medical Journal:*

Sir—At the February meeting of the Medical Society of the County of Erie, a resolution was unanimously adopted directing the president to appoint a committee to investigate and report upon the division of fees in the profession and the causes and remedies.

Dr. M. D. Mann's address on "The Division of Fees" was ordered printed in the BUFFALO MEDICAL JOURNAL and the Society was authorised and directed to send a reprint to each member of the profession in Erie County. The committee has been appointed by the president, Dr. Grover W. Wende and the consideration of the subjects has begun. You are requested to assist in the work and urged to send information, advice and suggestions concerning the topics mentioned or any other alleged abuses, which are in your opinion detrimental to the standing, ethics and ideals of the profession. If you have complaints, grievances or criticism of present conditions now is the time to present them, provided they can be controlled or ameliorated. With the object of securing definite, reliable data upon which to form reasonable conclusions, we have formulated the following questions and earnestly invite you to send an early reply.

1. What is your opinion regarding the extent of the practice of dividing fees or giving or receiving commissions?

2. Give any information concerning the methods employed.

3. Do you regard the division of fees as detrimental and dangerous to the medical profession and the public?

4. Do you favor the eradication of the practice and what means would you recommend?

---

1. This is the form of circular sent to the members of the Medical Society of the County of Erie. We print it here for the purpose of supplementing and accentuating the work of the committee.

5. What other forms of commercialism have come to your knowledge?

6. What is your position concerning contract work for societies, lodges, business organisations, factories, and the like? Give information from your experience.

7. Give facts and an expression of opinion relative to insurance examinations. The nature of the work and the fees.

8. What in your opinion are the chief causes of the recent invasion of commercialism and abuses in the medical profession?

9. State your position concerning the evils of overcrowding in the medical profession. Give your idea of the essential causes and what can be done to control and eradicate the unfortunate condition.

10. Give your ideas in reference to medical education, the superabundance of medical schools and their character with suggestions for reform.

11. Make suggestions concerning the proportionate and comparative fees obtained by the physician, surgeon and specialist. If you consider them unjust or unfair to the general practitioner what remedy would you recommend?

All communications will receive careful attention and will be treated as confidential by the committee if sent to the secretary of the committee, Dr. Wm. Gaertner, 194 East Utica Street.

John H. Pryor, Chairman

Grover W. Wende, Ex-officio

Bernard Bartow

Edwin A. Bowerman

Irving P. Lyon

Matthew D. Mann

F. Park Lewis

Thomas F. Welsh

William Gaertner, Secretary

---

Athletic Life in Relation to Degenerative Changes in the Cardiovascular System.—Robert E. Coughlin of Brooklyn, N. Y., considers that athletics which cause increase in the size of the heart are distinctly deleterious in later life, when the exercise is given up. Atrophy of the increased muscular elements leaves a weakened heart, which becomes a prey to myocarditis. Degenerative changes in the arteries and heart occur when the voluntary muscles lose their tone. Walking in the open air is the best form of exercise for these people. Athletic contests are physically dangerous, especially Marathon races. There is no excuse for the latter. There is great chance of injury to the heart in competitive games. From the twenty-first to the fortieth year of life the tendency is to take to drinking and the pleasures of the table. Sedentary habits lead to degenerative changes from lack of exercise and faulty metabolism resulting. More attention should be given to degenerative changes in the male vascular system after forty years of age. After middle life in athletes active exercise should be advised; once an athlete always an athlete.—*Medical Record*, April 2, 1910.

